# Conjunctival Lesion in a 9‐Year‐Old Boy

**DOI:** 10.1002/ccr3.71414

**Published:** 2025-11-23

**Authors:** Agata Anna Wykrota, Jessica Obst, Fidelis Flockerzi, Roman Saternus, Berthold Seitz, Fabian Norbert Fries

**Affiliations:** ^1^ Department of Ophthalmology South Tyrolean Health Service Bolzano Italy; ^2^ Department of Ophthalmology Saarland University Medical Center (UKS) Homburg/Saar Germany; ^3^ Institute of General and Special Pathology Saarland University Medical Center (UKS) Homburg/Saar Germany; ^4^ Department of Dermatology, Venereology, and Allergology Saarland University Medical Center (UKS) Homburg/Saar Germany

**Keywords:** ophthalmology, pathology and laboratory medicine, pediatrics and adolescent medicine, surgery

## Abstract

Conjunctival pseudolymphoma is a rare benign lymphoid proliferation that can mimic malignant lymphoma, especially in pediatric patients. Thorough histopathological, immunohistochemical, and molecular evaluation is essential for correct diagnosis and management, avoiding overtreatment.

## Graphical abstract

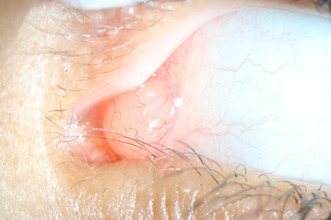

## 1 Introduction

Conjunctival pseudolymphoma, or conjunctival lymphoid hyperplasia, is a rare benign lymphoid proliferation of B‐cells and/or T‐cells. The exact mechanisms are not yet fully understood, but medications, foreign substances, and infections may play a role. Although it is considered a benign lesion, histopathological examination with supplementary immunohistochemistry and molecular pathology is essential to ensure a reliable distinction from malignant lymphoma, posing a diagnostic challenge. We present a case of a 9‐year‐old boy who presented with a painless conjunctival lesion in the left eye, which, after the histopathological examination, was confirmed to be a conjunctival pseudolymphoma.

## 2 Case History/Examination

**FIGURE 1:**
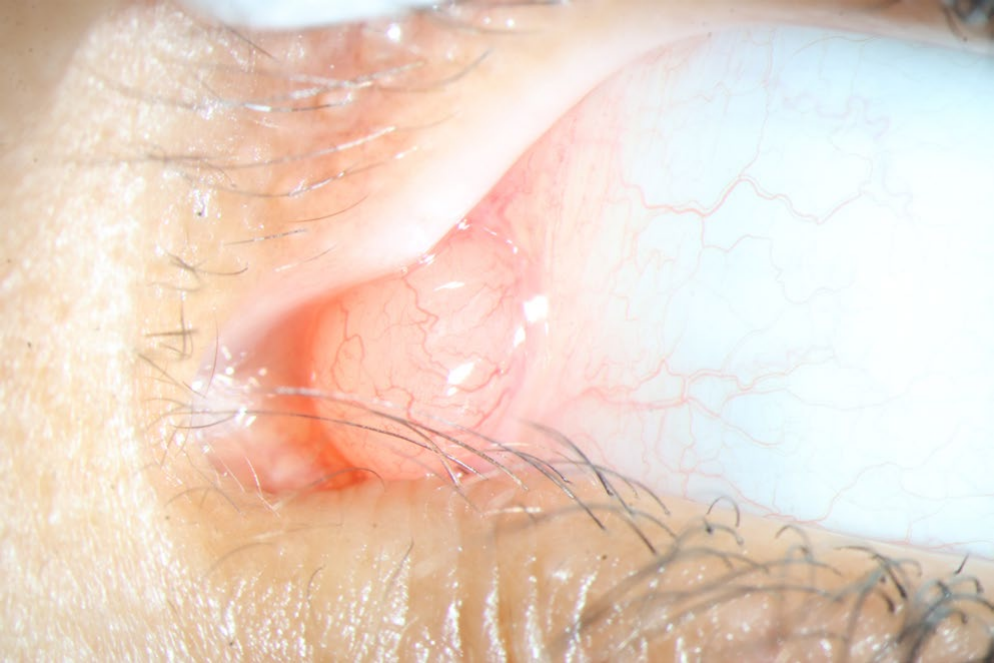
Conjunctival lesion in the inner canthus.

A nine‐year‐old boy presented with a painless conjunctival “cyst” in the left eye, persisting for several weeks. At the examination, a 1 × 1 cm, normally vascularized, salmon‐colored tumor with a shiny surface was found on the nasal conjunctiva bulbi of the affected eye, with involvement of the plica semilunaris (Figure 1). Ocular motility was free, the uncorrected visual acuity was 20/20 on both sides, and the preauricular lymph nodes were not enlarged. Medical history revealed neither chronic illnesses, systemic medication, prior trauma, nor recent or current infections. Due to a lack of improvement under local therapy with Isopto‐Max eye ointment (dexamethasone, neomycin sulfate, polymyxin B sulfate), excision of the conjunctival lesion was performed. First, we did a circular marking of the safety distance around the lesion using a surgical marker. Then, a sharp dissection and excision were performed using Vannas scissors and Colibri forceps without touching the lesion (no touch technique). After tumor removal, we changed the instruments and sutured the conjunctiva with Vicryl 8.0. This procedure was then followed by local therapy with Isopto‐Max eye ointment three times a day for a week and histopathological examination of the tissue.

## 3 Differential Diagnosis

Possible causes for conjunctival lesions/cysts may include inflammatory conditions, trauma, surgery, medications, foreign substances, neoplastic lesions, and infections.

## 4 Outcome and Follow‐Up

**FIGURE 2:**
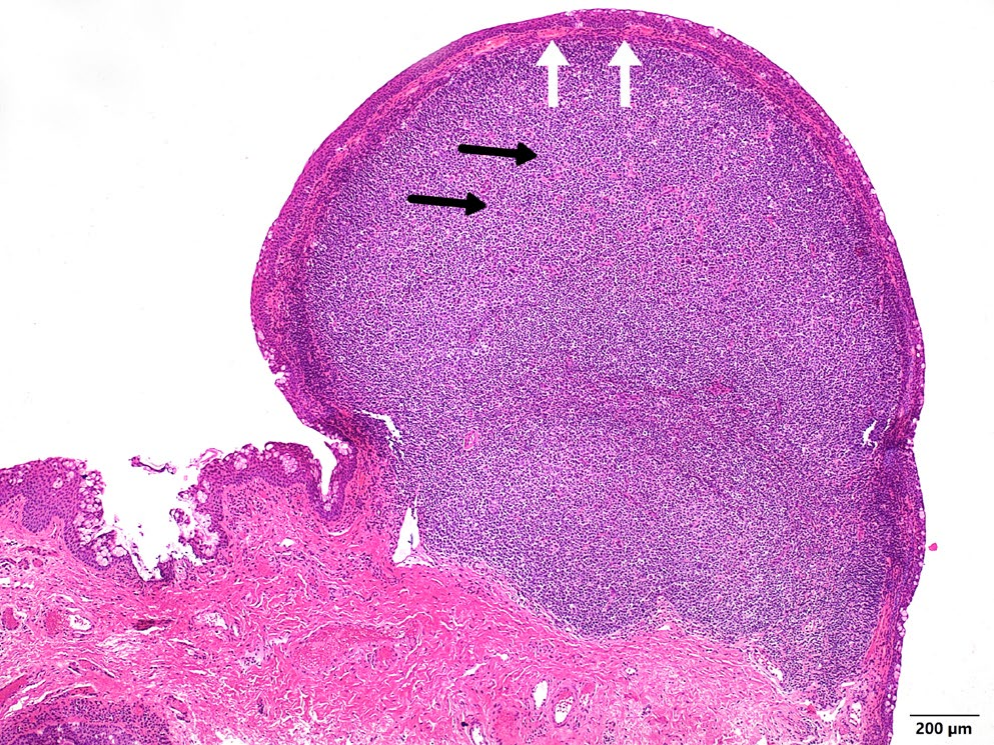
Hematoxylin–eosin staining (*HE staining, 40X magnification*) reveals a relatively well‐demarcated, monomorphic, small round blue cell infiltrate (*black arrows*) with a compressive growth pattern, without evidence of infiltration into adjacent structures (*white arrows*), particularly without evidence of lymphoepithelial lesions.

Histologically, a lymphoid lesion was identified, leading to a clinically visible protrusion of the conjunctival epithelium. Immunohistochemical analysis of the lesion revealed a quantitative predominance of CD20‐positive B‐lymphocytes and a few scattered CD3‐positive T‐lymphocytes. CD30‐positive blast cells were not detected, nor were atypical SOX10‐positive melanocytic cells. The hematoxylin–eosin staining (Figure 2) revealed a relatively well‐demarcated, monomorphic, small round blue cell infiltrate (*black arrows*) with a compressive growth pattern, without evidence of infiltration into adjacent structures (*white arrows*), particularly without evidence of lymphoepithelial lesions.

**FIGURE 3:**
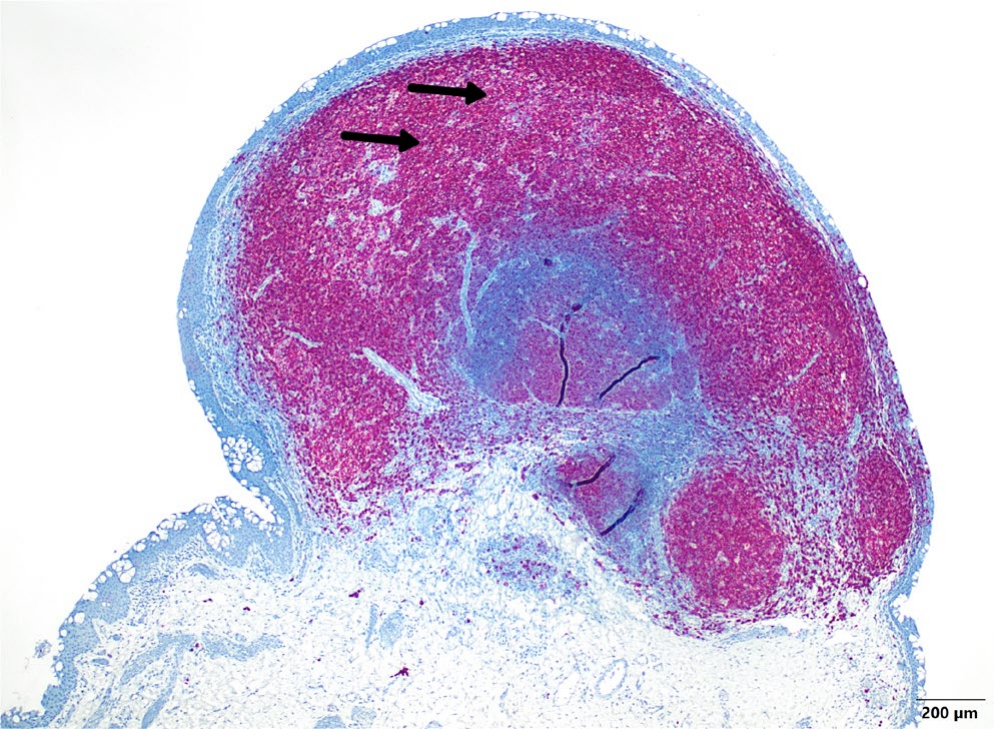
Immunohistochemical analysis reveals the mixed lymphoid infiltrate (CD3‐ and CD20‐positive), with a clear predominance of B‐lymphocytes (*black arrows, CD20‐IHC, 40X magnification*).

Subsequent immunohistochemical investigations characterized the lymphoid infiltrate as mixed‐cellular (CD3‐ and CD20‐positive) with a clear predominance of B‐lymphocytes (Figure 3, *black arrows*). A subsequent clonality analysis of the biopsied conjunctival tissue did not reveal a clonal B‐cell population.

Based on the described histological findings combined with the absence of monoclonality, a malignant lymphoma and an inflammatory juvenile compound nevus were ruled out, and the diagnosis of cutaneous, or, due to the specific location of the lesion, conjunctival lymphoid hyperplasia (so‐called pseudolymphoma) was established. The lesion was completely resected locally. In addition, further evaluation was performed, including a blood analysis and magnetic resonance imaging (MRI) of the cranium and orbit, which revealed no abnormalities. At both the initial presentation of the conjunctival lesion and subsequent follow‐ups up to one year after the excision, the patient remained healthy, with no signs of infection or other symptoms, as well as no local recurrence of the lesion. For safety, regular clinical monitoring was recommended.

## 5 Discussion

Depending on the localization, a cutaneous or conjunctival pseudolymphoma (or lymphoid hyperplasia) is understood as a benign lymphoid proliferation of B‐ and/or T‐cells [1]. It is one of the lymphoproliferative lesions of the ocular adnexa, which can occur in the conjunctiva, the eyelids, and the orbit, and is probably caused by antigen hyperstimulation. Conjunctival lymphoid hyperplasia rarely occurs in children and is predominantly found in patients around the age of 40. The exact mechanisms are not yet fully understood, but medications, foreign substances, and infections (e.g., *Borrelia burgdorferi* or mononucleosis) may play a role [2].

The most commonly cited categories of drugs causing pseudolymphoma are antihypertensives, anticonvulsants, monoclonal antibodies, and antidepressants [3]. This condition typically occurs in middle‐aged patients as pruritic papules, nodules, and plaques. There is almost no gender difference. Recent studies suggest that medications responsible for pseudolymphoma may impair immune function by disrupting immune surveillance, leading to abnormal lymphocyte proliferation, increased suppressor T‐cell activity, and hypogammaglobulinemia [4].

Although pseudolymphomas have mostly been documented as a consequence of ear piercing in adults, there are also reports of B‐cell pseudolymphomas in juvenile patients caused by ear piercing [5].

Borrelial pseudolymphoma, better known as borrelial lymphocytoma and previously referred to as *Lymphadenosis benigna cutis*, is a rare manifestation of Lyme disease that almost always occurs in children after infection with *Borrelia afzelii* [6]. Borrelial lymphocytoma presents as a solitary bluish‐red swelling with a diameter of up to several centimeters. The most common localization of borrelial lymphocytoma is the earlobe in children and the breast, near or at the nipple, in adults. The skin lesion is often accompanied by mild localized symptoms. Histologically, borrelial lymphocytoma is characterized by a dense polyclonal and predominantly B‐lymphocytic infiltration of the dermis and subcutis, often with germinal center formation [7].

Infectious mononucleosis mainly affects young male patients who present with painful cervical lymphadenopathy and sore throat. Blood analysis may show lymphocytosis and high titers of Epstein–Barr virus (EBV) IgG and IgM. A biopsy of the conjunctival lesion reveals a mixed infiltrate of lymphocytes, plasma cells, histiocytes, and cells with a prominent eosinophilic nucleus. Spontaneous regression of the lesion is expected in such cases [8].

Histopathological examination with supplementary immunohistochemistry and molecular pathology is essential to ensure a reliable distinction from malignant lymphoma [9].

In our patient, no active infectious disease was detected, leading to the conclusion of an idiopathic or foreign body‐induced lesion. Although studies show no transition of pseudolymphoma to lymphoma after a follow‐up of four years [10, 11], regular follow‐up checks should be carried out to rule out malignant lymphoma or local recurrence [12].

## 6 Conclusion

Conjunctival pseudolymphoma in children is a rare and still poorly described entity. In order to be able to make as accurate a diagnosis as possible and distinguish it from malignant lymphoma, a local resection should be performed, followed by histopathological examination with immunohistochemistry and molecular studies. At the last follow‐up, our patient remained healthy and showed no growth of the lesion one year following the complete excision, which speaks for the favorable prognosis of this type of conjunctival lesion.

## Author Contributions

**Agata Anna Wykrota:** conceptualization, data curation, formal analysis, investigation, methodology, visualization, writing – original draft, writing – review and editing. **Jessica Obst:** project administration, supervision, validation, writing – review and editing. **Fidelis Flockerzi:** conceptualization, data curation, methodology, project administration, supervision, validation, writing – review and editing. **Roman Saternus:** conceptualization, data curation, methodology, project administration, supervision, validation, writing – review and editing. **Berthold Seitz:** project administration, supervision, validation, writing – review and editing. **Fabian Norbert Fries:** conceptualization, data curation, methodology, project administration, supervision, validation, visualization, writing – review and editing.

## Consent

Written informed consent has been obtained from the patient and his parents.

## Conflicts of Interest

The authors declare no conflicts of interest.

## Data Availability

Data sharing not applicable to this article as no datasets were generated or analysed during the current study.

## References

[1] I. H. Wolf , L. Cerroni , R. Fink‐Puches , and H. Kerl , “The Morphologic Spectrum of Cutaneous Pseudolymphomas,” Journal der Deutschen Dermatologischen Gesellschaft 3 (2005): 710–720. 16173980 10.1111/j.1610-0387.2005.05536.x

[2] T. Ploysangam , D. L. Breneman , and D. F. Mutasim , “Cutaneous Pseudolymphomas,” Journal of the American Academy of Dermatology 38 (1998): 877–895. 9631994 10.1016/s0190-9622(98)70154-9

[3] I. Etesami , Y. Kalantari , S. Tavakolpour , H. Mahmoudi , and M. Daneshpazhooh , “Drug‐Induced Cutaneous Pseudolymphoma: A Systematic Review of the Literature,” Australasian Journal of Dermatology 64 (2023): 41–49. 36331821 10.1111/ajd.13951

[4] R. R. Pinheiro , B. Duarte , A. João , and A. Lencastre , “Cutaneous Pseudolymphoma Following Quadrivalent Human Papillomavirus Vaccination – A Rare Adverse Event,” Journal der Deutschen Dermatologischen Gesellschaft 16 (2018): 465–467. 10.1111/ddg.13470 29521018

[5] J. C. Slack , K. C. Kurek , F. O. G. Fraulin , and M. A. Brundler , “Cutaneous B‐Cell Pseudolymphoma (Lymphocytoma Cutis) of the Earlobe: A Poorly Recognized Complication of Ear Piercing in Children,” Fetal and Pediatric Pathology 5 (2020): 486–492. 10.1080/15513815.2020.1843573 33150803

[6] V. Woznica and I. Třešková , “Borrelial Pseudolymphoma of the Eyebrow in an Adult,” Rozhledy v Chirurgii 102 (2023): 88–90. 37185032 10.33699/PIS.2023.102.2.88-90

[7] G. P. Wormser , R. J. Dattwyler , E. D. Shapiro , et al., “The Clinical Assessment, Treatment, and Prevention of Lyme Disease, Human Granulocytic Anaplasmosis, and Babesiosis: Clinical Practice Guidelines by the Infectious Diseases Society of America,” Clinical Infectious Diseases 43 (2006): 1089–1134. 17029130 10.1086/508667

[8] C. W. Spraul , T. Mattfeldt , and G. K. Lang , “Conjunctival Pseudolymphoma in Infectious Mononucleosis,” Klinische Monatsblätter für Augenheilkunde 215 (1999): 68–69. 10448642 10.1055/s-2008-1034673

[9] D. Romero‐Pérez , M. Blanes Martínez , and B. Encabo‐Durán , “Cutaneous Pseudolymphomas,” Actas Dermo‐Sifiliográficas 107 (2016): 640–651. 27289134 10.1016/j.ad.2016.05.003

[10] S. E. Coupland , L. Krause , H. J. Delecluse , et al., “Lymphoproliferative Lesions of the Ocular Adnexa. Analysis of 112 Cases,” Ophthalmology 105 (1998): 1430–1441. 9709754 10.1016/S0161-6420(98)98024-1

[11] S. D. McLeod and D. P. Edward , “Benign Lymphoid Hyperplasia of the Conjunctiva in Children,” Archives of Ophthalmology 117 (1999): 832–835. 10369601 10.1001/archopht.117.6.832

[12] E. Domeier , R. Büttner , F. G. Holz , and K. U. Löffler , “Sudden Appearance of Conjunctival Tumor in a 7‐Year‐Old Boy,” Der Ophthalmologe 103 (2006): 616–619. 16328490 10.1007/s00347-005-1275-6

